# The Gut Bacterial Community of Mammals from Marine and Terrestrial Habitats

**DOI:** 10.1371/journal.pone.0083655

**Published:** 2013-12-30

**Authors:** Tiffanie M. Nelson, Tracey L. Rogers, Mark V. Brown

**Affiliations:** 1 Evolution and Ecology Research Centre, School of Biological, Earth and Environmental Sciences, University of New South Wales, Kensington, Australia; 2 Australian Institute of Marine Science, Water Quality and Ecosytem Health, Arafura Timor Research Facility, Casuarina, Australia; 3 Evolution and Ecology Research Centre; School of Biotechnology and Biomolecular Sciences, University of New South Wales, Kensington, Australia; Argonne National Laboratory, United States of America

## Abstract

After birth, mammals acquire a community of bacteria in their gastro-intestinal tract, which harvests energy and provides nutrients for the host. Comparative studies of numerous terrestrial mammal hosts have identified host phylogeny, diet and gut morphology as primary drivers of the gut bacterial community composition. To date, marine mammals have been excluded from these comparative studies, yet they represent distinct examples of evolutionary history, diet and lifestyle traits. To provide an updated understanding of the gut bacterial community of mammals, we compared bacterial 16S rRNA gene sequence data generated from faecal material of 151 marine and terrestrial mammal hosts. This included 42 hosts from a marine habitat. When compared to terrestrial mammals, marine mammals clustered separately and displayed a significantly greater average relative abundance of the phylum *Fusobacteria*. The marine carnivores (Antarctic and Arctic seals) and the marine herbivore (dugong) possessed significantly richer gut bacterial community than terrestrial carnivores and terrestrial herbivores, respectively. This suggests that evolutionary history and dietary items specific to the marine environment may have resulted in a gut bacterial community distinct to that identified in terrestrial mammals. Finally we hypothesize that reduced marine trophic webs, whereby marine carnivores (and herbivores) feed directly on lower trophic levels, may expose this group to high levels of secondary metabolites and influence gut microbial community richness.

## Introduction

Bacteria inhabiting the gastro-intestinal tract of mammals expand their host’s metabolic potential by harvesting energy that would otherwise be inaccessible [1]–[3]. This symbiosis between mammals and bacteria has contributed, in part, to the success of the class *Mammalia,* allowing them to radiate in large numbers to occupy a variety of environmental niches [3], [4]. Herbivorous mammals, for instance, were able to survive on plant material after acquiring a gut bacterial community with the capability to digest cellulose in plant cell walls [5].

Mammalian hosts first acquire their gut bacterial community during transport through the birth canal and subsequently through maternal, social and environmental transmissions [6]–[8]. Genetic factors within the host also shape the gut bacterial community, a result of their long history of co-evolution [9]–[11]. This is evident in the strong physiological effects which the gut bacterial community can exert on the host mammal, such as modulating the immune response system or affecting brain development [12], [13].

In a pioneering study, Ley et al. 2008 [3] compared the faecal bacterial community of a variety of terrestrial mammals and identified that host phylogeny, diet and, to a lesser extent, gut morphology influenced the composition of the gut bacterial community. Since then, studies have further confirmed that the composition of the gut bacterial community follows along evolutionary lineages [11], [14]. Recently, diet has been shown to be the primary driver of functional capacity in the gut, resulting in a convergence of microbial communities between phylogenetically un-related hosts [15].

Further insight could be gained by comparing a diverse range of extant mammals with differing life history traits. One group of mammals that have been relatively understudied are marine mammals. Their comparatively recent evolution and differing life history traits and adaptation to a marine habitat [16], suggest they are a necessary addition to the current understanding of drivers shaping the mammalian gut bacterial community.

To understand patterns in gut bacterial communities, the faecal bacterial communities of a broad range of terrestrial and marine mammals were compared. Marine mammals included two species of seals inhabiting the Antarctic [17]; three species of seals inhabiting the Arctic [18] and data from one dugong (a marine herbivore) [19]. Terrestrial mammals included carnivores, omnivores and herbivores from a range of phylogenetic groups (number of individuals, n = 109). The aim of this study was to identify broad scale patterns of gut bacterial communities of mammals from marine and terrestrial habitats.

## Materials and Methods

### Ethics Statement

Samples collected from southern elephant seals and leopard seals were carried out in strict accordance with the recommendations in the Australian Code of Practice for the Care and Use of Animals for Scientific Purposes. Protocols used in the study were approved by the University of New South Wales Animal Care and Ethics Committee (permit number 08/83B and 03/103B). The southern elephant seal is listed as vulnerable under the Environment Protection and Biodiversity Conservation Act 1999 (EPBC Act) and listed under the Convention on International Trade in Endangered Species of Wild Fauna and Flora (CITES). Permission to export southern elephant seal biological materials was obtained from the Australian Government Department of the Environment, Water, Heritage and the Arts (permit number 2008-AU-534289).

Permission to access regions in Antarctica where seals were located was approved by the Ministry of Foreign Affairs and the Dirección Nacional del Antártico. Southern elephant seals in this study were located in Antarctic Special Protected Area 132 ‘Peninsula Potter’ and additional permissions were obtained through the Dirección Nacional del Antártico under Article 3, Annex II of the Madrid Protocol to the Antarctic Treaty (no permit number). For southern elephant seal males and leopard seals, individuals were anaesthetised using a mixture of tiletamine and zolazepam (Zoletil – Virbac Australia) at a combined dose of 1 mg/kg. Female southern elephant seals were anaesthetised with 3–6 mg/kg ketamine hydrochloride. On all occasions, procedures were performed by qualified personnel and all efforts were made to minimize suffering.

### Selection of Studies for Comparison

Studies for comparison were selected on the basis that analysis methods for bacterial community composition sequenced a region of the 16S rRNA gene using highly conserved bacterial or universal primer sets. The specific methods each study used to generate this data are outlined in Table S1.

Individual sequence data were obtained from the National Centre for Biotechnology Information website (http://www.ncbi.nlm.nih.gov/) or directly from locations specified in source articles. The dataset comprising Antarctic seal gut bacterial community by the same authors is deposited to the database Dryad (www.datadryad.org) under the provisional DOI:10.5061/dryad.42f2q [20]. In total the combined dataset contained samples from 151 individual mammal hosts.

### Preparation of Datasets for Comparison

Sequence taxonomy was assigned using the Ribosomal Database Project II (RDP) v.10 Classifier tool [21]. Taxonomy of sequences was assigned using RDPs Naïve Bayesian Classifier which classifies sequences to the genus level [21]. As the total number of sequences in each dataset differed, datasets were randomly sub-sampled to a maximum of 100 sequences using the software Daisy-Chopper (www.genomics.ceh.ac.uk/GeneSwytch/). The result was a total of 13,848 sequences from 151 mammalian hosts (Table S2).

Meta-analyses such as this may be prone to study effects, whereby similarity or differences in methodology, rather than ecology, generate the observable patterns. To address concerns over any potential study effects, we selected and analysed data from four host phylogenetic families, the *Phocidae, Ursidae, Hominidae* and *Canidae*, (Figure S1) as each contained samples from different studies. When analysed in the same manner as the main dataset (see methods) the gut bacterial community of the host cluster more closely based on phylogenetic family of the host (Figure S1A) than by which study the host originated from (Figure S1B, Table S1).

In addition, to examine the effect of data standardisation versus data rarefaction (to enable incorporation of datasets that contained fewer than 100 sequences per host), the complete dataset was rarefied to 24 sequences per host, which was the lowest number present in any study used. When analysed using the same techniques as described below (see methods) this subsampled dataset generated the same statistically significant overall patterns as those observed when using the expanded dataset (see Table S2 and Figure S2).

The assessments of study effects give provide the author’s with confidence that the results reported herein describe ecological effects rather than methodological effects.

### Statistical Analyses of Data

The combined dataset was standardised prior to transformation. This involved converting abundance counts into relative percentages for each individual host. A Bray-Curtis dissimilarity matrix [22] was generated from square-root transformed data. To facilitate comparisons of the gut bacterial community between hosts, hosts were assigned to *a priori* groups based on habitat (marine or terrestrial), phylogenetic family, dominant dietary source (carnivorous, omnivorous or herbivorous) or gut morphology (simple, hind or foregut fermenters). These factors have been identified previously as potential drivers of gut bacterial community composition [3], [15]. Similarities between hosts and host groups were visualised using non-metric multi-dimensional scaling (nMDS) [23]. The result of nMDS ordination is a two-dimensional plot where the position of each sample is determined by its distance from all other points in the analysis. The contribution of classified genera to the observed dissimilarity between groups in the nMDS were calculated using SIMPER (similarity percentages procedure) [24]. SIMPER decomposes average Bray-Curtis dissimilarities between all pairs of hosts into percentage contributions from each classified genus and therefore identifies which genera are characteristic of bacterial community structure [24]. The non-parametric estimator of species richness, Chao1 [25], was calculated using genus abundance data for each host using the online software program EstimateS [26].

Differences in the composition of the gut bacterial community between hosts were tested with the non-parametric permutation procedure ANOSIM (Analysis of Similarity) as it is more robust to heterogeneous dispersion of data [24]. ANOSIM is applied to the dissimilarity matrix and generates a test statistic, R. The magnitude of R is indicative of the difference within groups and between groups and is scaled to lie between −1 and +1, a value of zero represents the null hypothesis (no difference between groups) and value towards one represents the alternative hypothesis (all similarities within groups are less than any similarity between groups) [24]. R-values >0.50 (**) were interpreted as well separated; R >0.30 (*) as overlapping but clearly different; and, R >0.20 as barely separable at all. Results were considered significant where *P-value* = <0.025. Significant differences in bacterial richness and phyla abundance between groups were tested using a Student’s T-Test in Excel 2010 (Microsoft Pty Ltd). All other statistical tests were performed using the software PRIMER-E v6 [24].

## Results

### Patterns in the Gut Bacterial Community of Marine and Terrestrial Mammals

**Figure 1:**
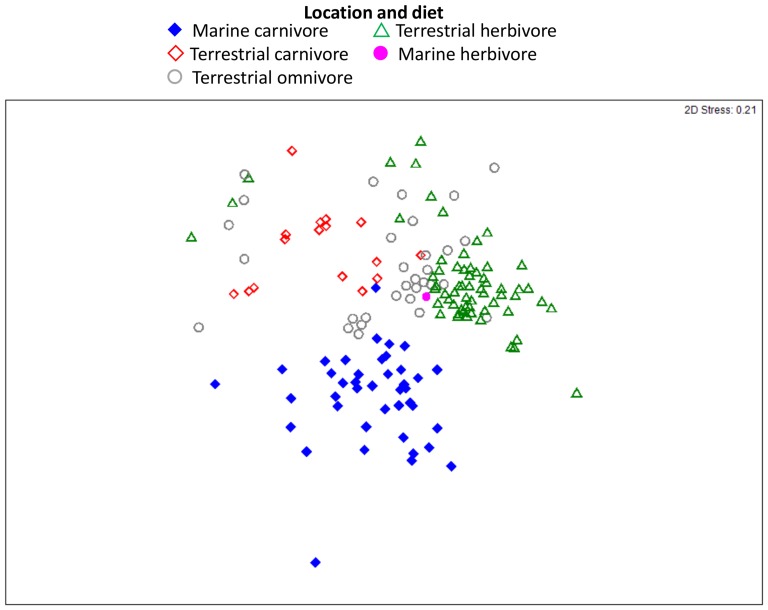
Influence of diet and habitat on the gut bacterial community of mammals. nMDS ordination plot dislaying similarity of the gut bacterial community in the host mammal as grouped by diet and habitat. See Figure S4 for detailed display of this figure.

Herbivores and carnivores displayed significant differences in the composition of their gut bacterial communities (ANOSIM: R = 0.49, *p* = <0.01; Table 1; Figure 1). The gut bacterial community of marine carnivores and terrestrial carnivores was significantly different (R = 0.69, *p* = <0.01; Table 1). Con-specific hosts from the same family were more similar than non-con-specific hosts (R = 0.50, *p* = <0.01). Across all mammals, the gut morphology of hosts did not contribute to significant differences in the gut bacterial community (Table 1). Previously, the influence of captivity in leopard seals was identified as a strong driver of the gut bacterial community (see Nelson et al. 2012) [17]. However, in this study, six host species were sampled from both captive and wild habitats and compared in an nMDS plot (Figure S3) and it was clear that captive and wild con-specifics clustered closer to one another than they did to unrelated hosts.

**Table 1:** Difference of the gut bacterial community of host mammals based on groupings of diet, habitat, phylogeny, and gut morphology using ANOSIM.

Source of variation	Pair-wise comparisons	*R*	*p*
Diet		0.39*	<0.01
	Herbivores, omnivores	0.33	<0.01
	Herbivores, carnivores	0.49*	<0.01
	Omnivores, carnivores	0.26	<0.01
Diet and habitat		0.52**	<0.01
	Terrestrial herbivores,terrestrial omnivores	0.33*	<0.01
	Terrestrial herbivores,terrestrial carnivores	0.62**	<0.01
	Terrestrial herbivores,marine carnivores	0.65**	<0.01
	Terrestrial omnivores,terrestrial carnivore	0.32	<0.01
	Terrestrial omnivores,marine carnivores	0.50**	<0.01
	Terrestrial carnivores,marine carnivores	0.69**	<0.01
Phylogenetic order		0.19	<0.01
Phylogenetic family		0.50**	<0.01
Gut morphology		0.11	<0.01
	Hindgut fermenters,simple guts	0.14	<0.01
	Hindgut fermenters,foregut fermenters	0.17	<0.01
	Simple guts,foregut fermenters	0.15	<0.01

ANOSIM of gut bacterial abundance data was used to generate a permutated Global R statistic (R) and permutated p-value (*p*). Significance level: **R = >0.5, *R = 0.3< R <0.5. Comparison of these results with rarefied data is displayed in Table S3.

### Taxonomic Differences in Gut Bacterial Community Composition between Marine and Terrestrial Mammals

**Figure 2:**
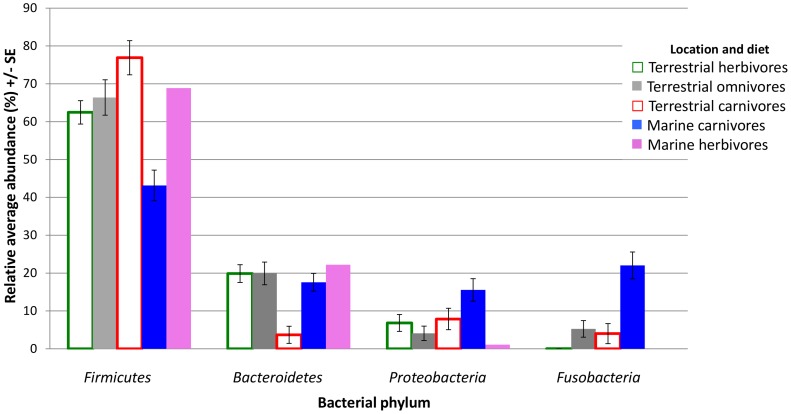
Differences in abundance of dominant phyla in the gut bacterial community of mammals based on groupings of habitat and diet. Average relative abundance of each major phyla in the gut bacterial community of host mammals grouped by habitat and diet. Error bars represent standard errors (SE). The lack of replication in the marine herbivore grouping does not allow for estimation of SE or significance testing. Student’s paired t-test were conducted between groups as displayed in Table 2.

Marine carnivores possessed a significantly lower average relative abundance of the phylum *Firmicutes* in their gut bacterial community with 43.2±4.0 compared to 65.6±2.3% in the gut bacterial community of terrestrial mammals (Student’s paired t-test *p = *<0.001; Table 2 and Figure 2) and 68.9% in the gut bacterial community of the marine herbivore (Figure 2). The phylum *Proteobacteria* was significantly more abundant in marine carnivores with an average relative abundance of 15.6±3.0% compared with 5.9±1.6% in the gut bacterial community of terrestrial omnivores and herbivores (t-test *p = *0.004 and 0.018, respectively; Table 2 and Figure 2). The phylum *Bacteroidetes* was similar for each of the dietary groups of terrestrial and marine mammals with an average relative abundance of 19.2±1.8% with the exception of terrestrial carnivores, which displayed a significantly reduced abundance of 3.7±2.3% (t-test *p* = <0.002; Table 2 and Figure 2).

**Table 2:** Differences in presence of dominant phyla in the gut bacterial community of mammals based on groupings of habitat and diet using Student’s paired t-test.

Comparison	*P*			
	*Firmicutes*	*Bacteroidetes*	*Proteobacteria*	*Fusobacteria*
Terrestrial herbivores,terrestrial omnivores	0.473	0.996	0.423	0.001**
Terrestrial herbivores,terrestrial carnivores	0.033	0.001**	0.828	0.002**
Terrestrial herbivores,marine carnivores	<0.001***	0.502	0.018*	<0.001***
Terrestrial omnivores,terrestrial carnivores	0.165	0.001**	0.266	0.735
Terrestrial omnivores,marine carnivores	<0.001***	0.536	0.004**	<0.001***
Terrestrial carnivores,marine carnivores	<0.001***	0.002**	0.151	0.005**
Terrestrial mammals,marine mammals(all diet types)	<0.001***	0.991	0.002**	<0.001***

Student’s paired t-test of gut bacterial abundance data to generate a p-value (*p*). Significance level: *p* = ≤0.001 (***), *p* = 0.01 (**), *p* = 0.025 (*). The lack of replication in the marine herbivore grouping does not allow for significance testing.

**Figure 3:**
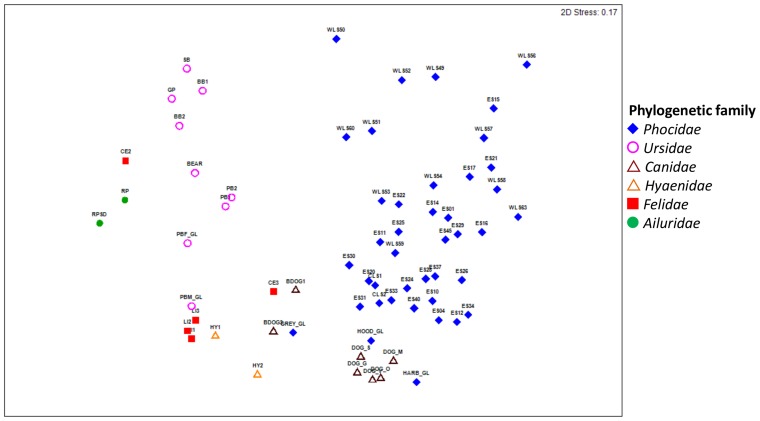
Influence of phylogenetic family on the gut bacterial community of mammals from the order *Carnivora*. nMDS plot displays gut bacterial community of all host mammals in the order *Carnivora* grouped by family. Host species labels are as follows: (RP) red panda; (BB) black bear; (PB) polar bear; (SB) spectacled bear; (GP) giant panda; (BEAR) bear from Norway; (CE) cheetah; (BDOG) bushdog; (DOG) domestic dog; (LI) lion; (HY) spotted hyena; (GREY) grey seal; (HOOD) hooded seal; (HARB) harbour seal; (ES) southern elephant seal; (WLS) leopard seal.

The phylum *Fusobacteria* was significantly greater in the gut bacterial community of marine carnivores with an average relative abundance of 22.0±3.5% compared with 2.1±0.8% in other dietary groups (t-test *p* = <0.001; Figure 2 and Table 2). Domestic dogs, *Canis lupus familiaris*, were the only non-marine carnivore with a higher than average abundance of the phylum *Fusobacteria* with 32.7±1.6%. The domestic dog clustered closest to the marine carnivores in the nMDS plot (Figure 1 and S4) and this is further highlighted when observing the plot with only members of the order *Carnivora* (Figure 3).

Members of the family *Ursidae* are also seen to cluster closely together regardless of dietary preference, which includes herbivores, omnivores and carnivores (Figures 3 and S4). Overlap in the presence of particular genera between host dietary groups was evident. Marine and terrestrial herbivores, as well as omnivores, displayed overlap in the genera *Anaerotruncas*, *Ruminococcus* and *Roseburia* from the phylum *Firmicutes* (Table S4). These were less abundant in marine or terrestrial carnivores. Some genera, including the genus *Oscillibacter* from the phylum *Firmicutes*, and the genera *Prevotella* and *Bacteroides* from the phylum *Bacteroidetes* were abundant in all groups except for terrestrial carnivores (Table S4). Likewise, some genera, such as *Coprococcus* and *Blautia* from the phylum *Firmicutes* were abundant across all groups with the exception of the marine carnivores. The genus *Lactobacillus* from the phylum *Firmicutes* was commonly shared between carnivorous hosts compared with other dietary groups (Table S4).

### Richness of Mammal Guts

**Figure 4:**
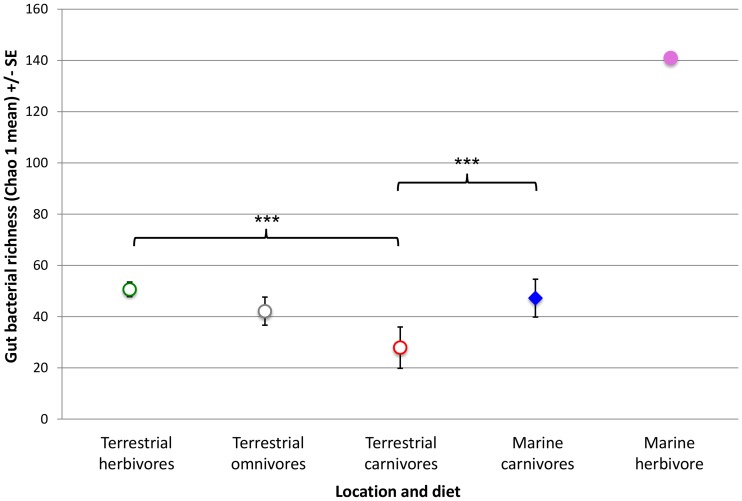
Richness of the gut bacterial community of mammals grouped by habitat and diet. Richness of the gut bacterial community was measured using Chao 1 mean. Error bars represent standard error (SE). Student’s paired t-test were conducted between groups with significance level: *p* = ≤0.001 (***), *p* = 0.01 (**), *p* = 0.025 (*). The lack of replication in the marine herbivore grouping does not allow for estimation of SE or significance testing.

Herbivores possessed a faecal bacterial community significantly richer than that of carnivores or omnivores (Figure 4). The single marine herbivore displayed a gut bacterial community richer (Chao 1 mean = 141.0) than that of terrestrial herbivores (Chao 1 = 51.0±2.8) or marine carnivores (Chao 1 = 47.0±7.4), although insufficient replication did not allow for significance testing of this pattern (Figure 4). The marine carnivores possessed significantly richer faecal bacterial community than the terrestrial carnivores (Figure 4). Additionally, hindgut fermenters displayed a significantly richer gut bacterial community than hosts with simple gut morphology (t-test: *p* = 0.0028).

## Discussion

### Comparative Marine and Terrestrial Mammals Gut Bacterial Communities

The composition of the gut bacterial community of the marine mammals available at the time of this study is clearly distinct from that of the available terrestrial mammals. Differences in the gut bacterial community of carnivorous marine mammals appears to be due, in part, to their considerably reduced abundance of *Firmicutes* and increased abundance of *Fusobacteria* compared to terrestrial mammals. Members of the phylum *Fusobacteria* range from facultative anaerobes to obligate anaerobes that ferment carbohydrates or amino acids to produce various organic acids including acetic, formic and butyric acid [27]–[29]. Species from the phylum *Fusobacteria* occur in a range of habitats, including sediments as well as the oral or intestinal habitats of animals [28], [30]–[32]. Future functional analysis will provide insight as to the specific roles of these phyla in the represented hosts.

The occurrence of greater than average abundance of *Fusobacteria* within the gut of dogs and also within the gut of marine carnivores suggests an interesting trend (see Figure S4). The *Canidae* (dogs) are located with the *Phocidae* in the order *Carnivora* [33] (see Figure S5). Canids and phocids possess shared immune system receptors and diseases, such as *Morbillivirus*, that are capable of passing between dog and seal hosts [34], [35]. Although it is beyond the scope of this study to make conclusions about the strength of this pattern of shared *Fusobacteria*, future investigations may help to understand if evolutionary links between seals and dogs can be identified through host-associated microbes.

Composition of the leopard seal gut bacterial community was previously shown to differ significantly in captivity compared with wild hosts, as a result of specific dietary items and local habitat differences [17]. However, this study suggests that the influence of captivity is reduced when comparisons are made at a broader scale (see Figure S3). Host phylogeny and dietary types were stronger indicators of gut bacterial community similarity than was captivity. However, it seems apparent that captive and wild con-specific hosts do display differences in their gut bacterial community at a finer scale. Specific dietary items, antibiotic administration and local exposures result in altered gut bacterial communities in captive individuals [15], [36], [37] and it is likely these factors that are contributing to the observed differences in the wild and captive hosts compared in this study.

Several challenges are faced when consuming plant material as a primary food source due to the indigestible cell walls [5]. The need to access complex carbohydrates in plants, such as cellulose and starch, is thought to be the driver of a rich gut bacterial community of herbivores [3]. The diversity of gut bacterial communities associated with terrestrial herbivores has been previously identified as significantly richer than those of terrestrial omnivores and carnivores [3]. This finding is also supported by our data. Further, our data indicate that marine carnivores have a richer gut bacterial community than terrestrial carnivores, whilst the one marine herbivore sampled had the richest bacterial community of all terrestrial herbivores. Taken together these results suggest that mammals living in marine habitats may generally possess a richer gut bacterial community than their diet-equivalent terrestrial mammals. In the marine environment, secondary metabolites produced by plants and other primary producers may be considerably higher [5], [38], [39], which could impact the gut bacterial community of herbivores or carnivores at higher trophic levels. Dugongs primarily consume seagrass and are known to occasionally supplement their diet with macro invertebrates and algae [40], [41]. The dugong sampled in this case was in captivity during sampling and had been fed a diet of the eelgrass, *Zostera marina* [19]. Eelgrass has been identified as producing secondary chemical defences, specifically phenolic acids, which have the capacity to cause considerable reduction of bacteria at even low dosages in experimental studies [42], [43]. The marine carnivores in this study are also known to feed directly on lower trophic levels, causing them to be exposed to secondary metabolites [44], [45]. One of the consequences of these different traits specific to marine based diets is that marine mammals may require a gut bacterial community with a greater diversity of functions enabling the breakdown of excess chemical compounds. Increased sampling of marine herbivores would enable us to unravel these processes.

## Supporting Information

Figure S1 **Mammals in families from different studies.** To display the impacts of possible study effects due to the different techniques used across studies, these nMDS plots display relationships between gut bacterial communities generated using different methods from the phylogenetic families *Phocidae, Canidae, Ursidae* and *Hominidae* labelled by family (A) and by study (B).
(TIF)

Figure S2 **Repeat figures from study with dataset rarefied to minimum number sequences per host.** Figure 1 (A) and Figure 3 (B) are repeated here to show the similarity in structure when using the minimum number of 24 rarefied sequences per host.
(TIF)

Figure S3 **Similarity of the gut bacterial community of host mammals with captive and wild representatives.** Non-metric multidimensional scaling ordination plot displays similarity of the gut bacterial community of host mammals with representatives from captive (c) and wild (w) habitats.
(TIF)

Figure S4 **Similarity of the gut bacterial community of mammals grouped by diet and habitat.** Detailed nMDS ordination plot of Figure 1 with host labels and enlarged region for clarity.
(TIF)

Figure S5 **Descriptive phylogeny of families from the order** ***Carnivora*** **.** Adapted from Agnarsson *et al.* 2010 [33]. Members of the *Phocidae* are marked with red circle.
(TIF)

Table S1 Overview of main methods employed by included studies.
(DOCX)

Table S2 Characteristics of mammalian hosts used in the study. Abbreviated table data is as follows: number of sequences used (No. of seq.); gut morphology (Gut morph.); hindgut fermenter (HG); foregut fermenter (FG); simple gut (S); marine (M); terrestrial (T); carnivore (C); herbivore (H); and omnivore (O).
(DOCX)

Table S3 Comparison of ANOSIM results between the genera rarefied to the minimum of 24 and those subsampled to 100. Results display those reported in Table 1 and the comparable results when the dataset was rarefied to the minimum number of sequences represented by any one host which was 24. ANOSIM of gut bacterial abundance data was used to generate a permutated Global R statistic (R) and permutated p-value (p). Significance level: **R = >0.5, *R = 0.3< R <0.5.
(DOCX)

Table S4 Characteristic genera in the gut bacterial community of mammal hosts grouped by diet and habitat. The foremost ten characteristic genera in the gut bacterial community of hosts identified using SIMPER analysis. Hosts are grouped based on diet and habitat.
(DOCX)
