# Correction: The Gut Bacterial Community of Mammals from Marine and Terrestrial Habitats

**DOI:** 10.1371/journal.pone.0099562

**Published:** 2014-05-30

**Authors:** The PLOS ONE Staff

There is an error in affiliation for author Tracey L. Rogers. The correct affiliation is: Evolution & Ecology Research Centre, School of Biological, Earth & Environmental Sciences, University of New South Wales.

## References

[1] Nelson TM, Rogers TL, Brown MV (2013) The Gut Bacterial Community of Mammals from Marine and Terrestrial Habitats. PLoS ONE 8(12): e83655 doi:10.1371/journal.pone.0083655 24386245 10.1371/journal.pone.0083655 PMC3875473

