# Supplementary figures and images for: The Gut Bacterial Community of Mammals from Marine and Terrestrial Habitats
Source: PLoS One. 2013 Dec 30;8(12):e83655. doi: 10.1371/journal.pone.0083655 (PMC3875473; doi:10.1371/journal.pone.0083655)

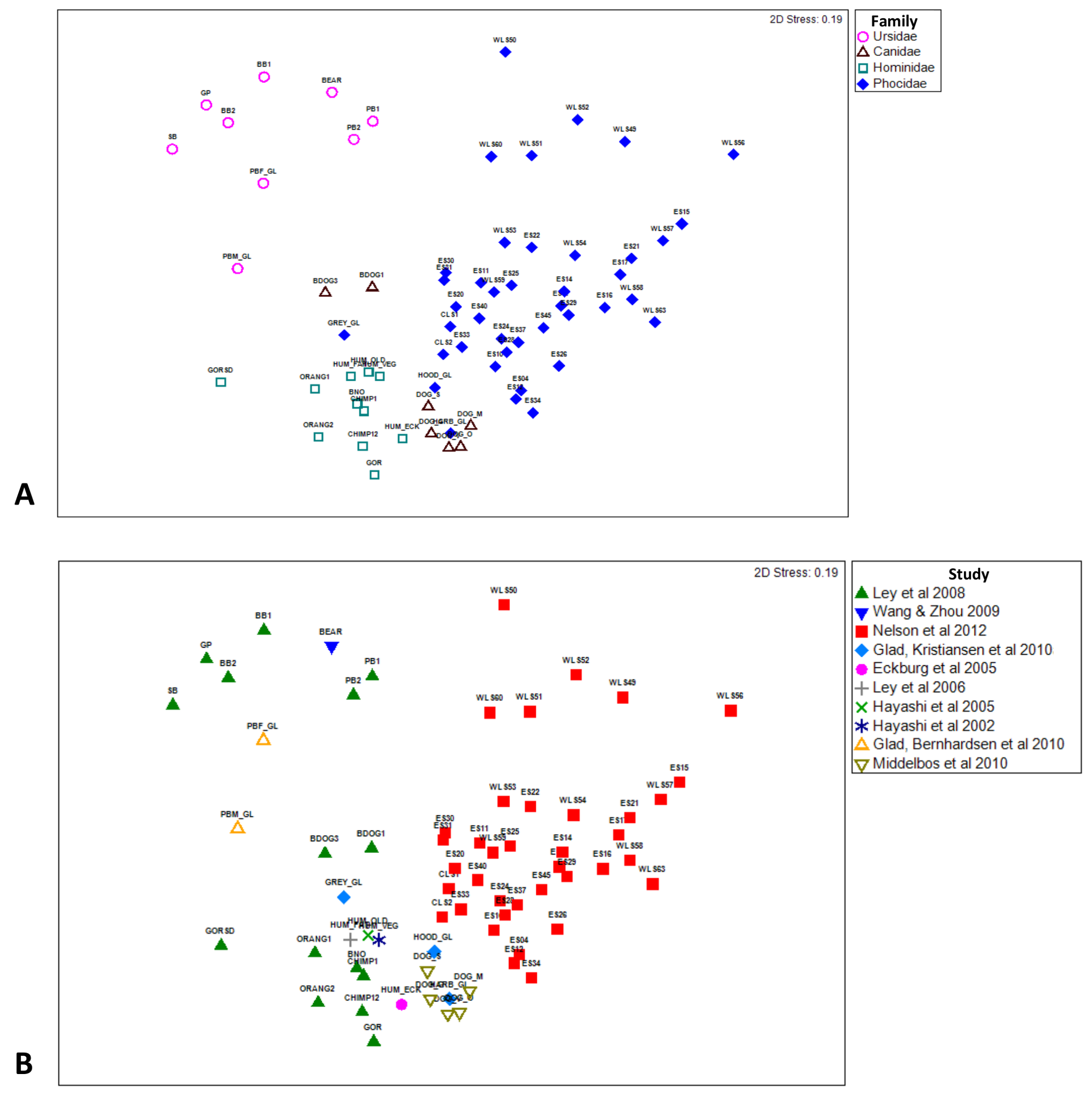

Supplement: Figure S1 — Mammals in families from different studies. To display the impacts of possible study effects due to the different techniques used across studies, these nMDS plots display relationships between gut bacterial communities generated using different methods from the phylogenetic families Phocidae, Canidae, Ursidae and Hominidae labelled by family (A) and by study (B). (TIF) [file pone.0083655.s001.tif]

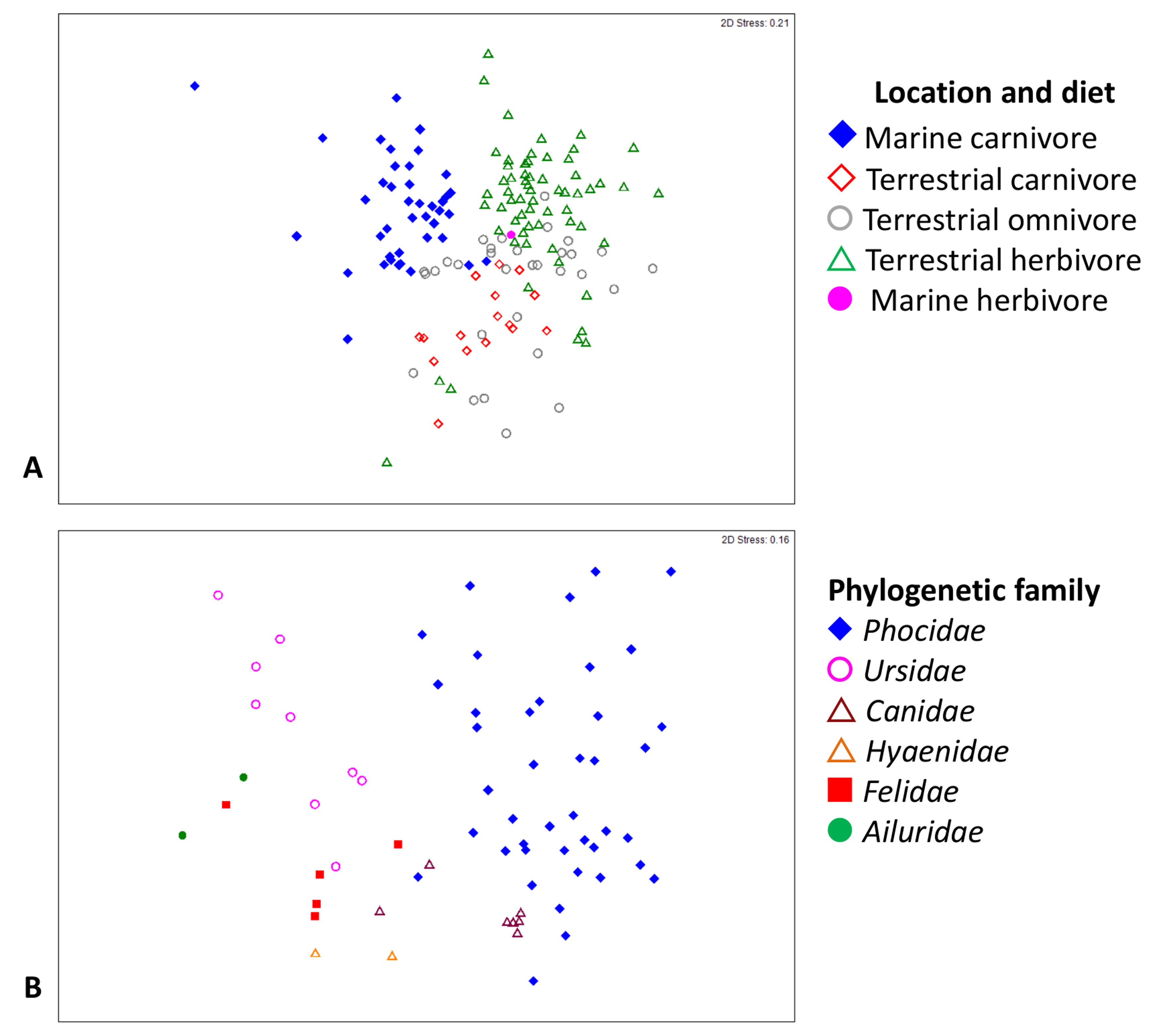

Supplement: Figure S2 — Repeat figures from study with dataset rarefied to minimum number sequences per host. Figure 1 (A) and Figure 3 (B) are repeated here to show the similarity in structure when using the minimum number of 24 rarefied sequences per host. (TIF) [file pone.0083655.s002.tif]

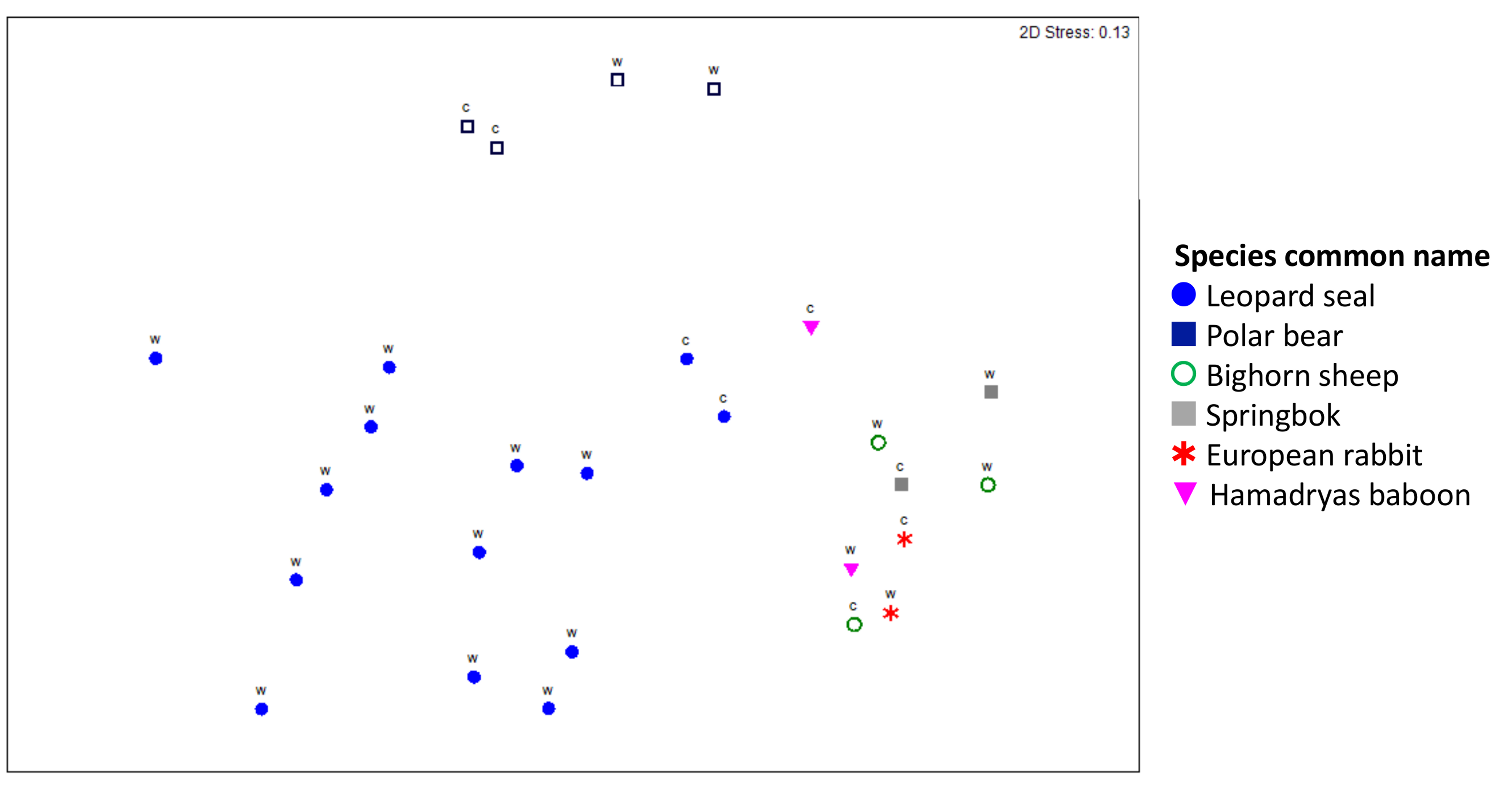

Supplement: Figure S3 — Similarity of the gut bacterial community of host mammals with captive and wild representatives. Non-metric multidimensional scaling ordination plot displays similarity of the gut bacterial community of host mammals with representatives from captive (c) and wild (w) habitats. (TIF) [file pone.0083655.s003.tif]

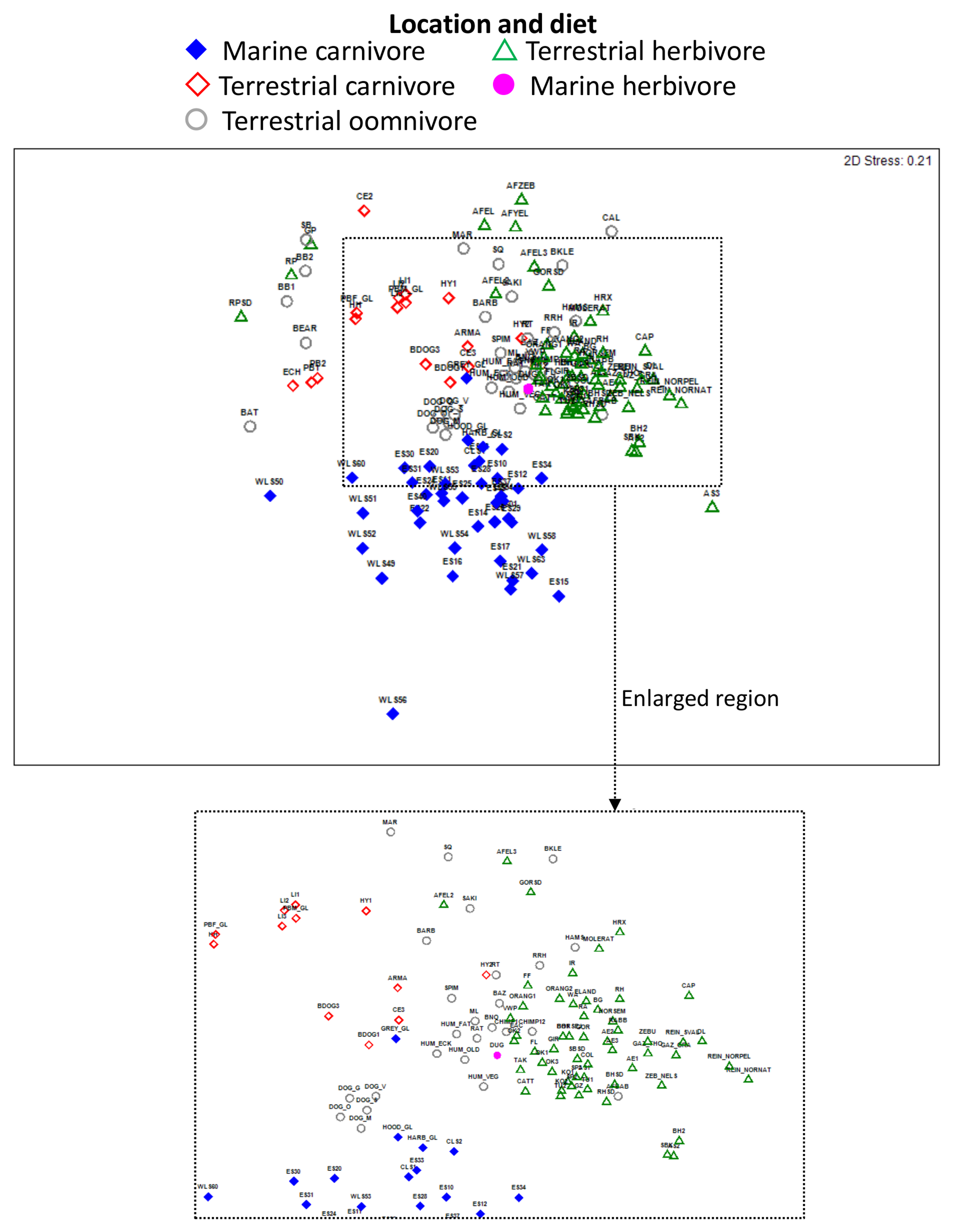

Supplement: Figure S4 — Similarity of the gut bacterial community of mammals grouped by diet and habitat. Detailed nMDS ordination plot of Figure 1 with host labels and enlarged region for clarity. (TIF) [file pone.0083655.s004.tif]

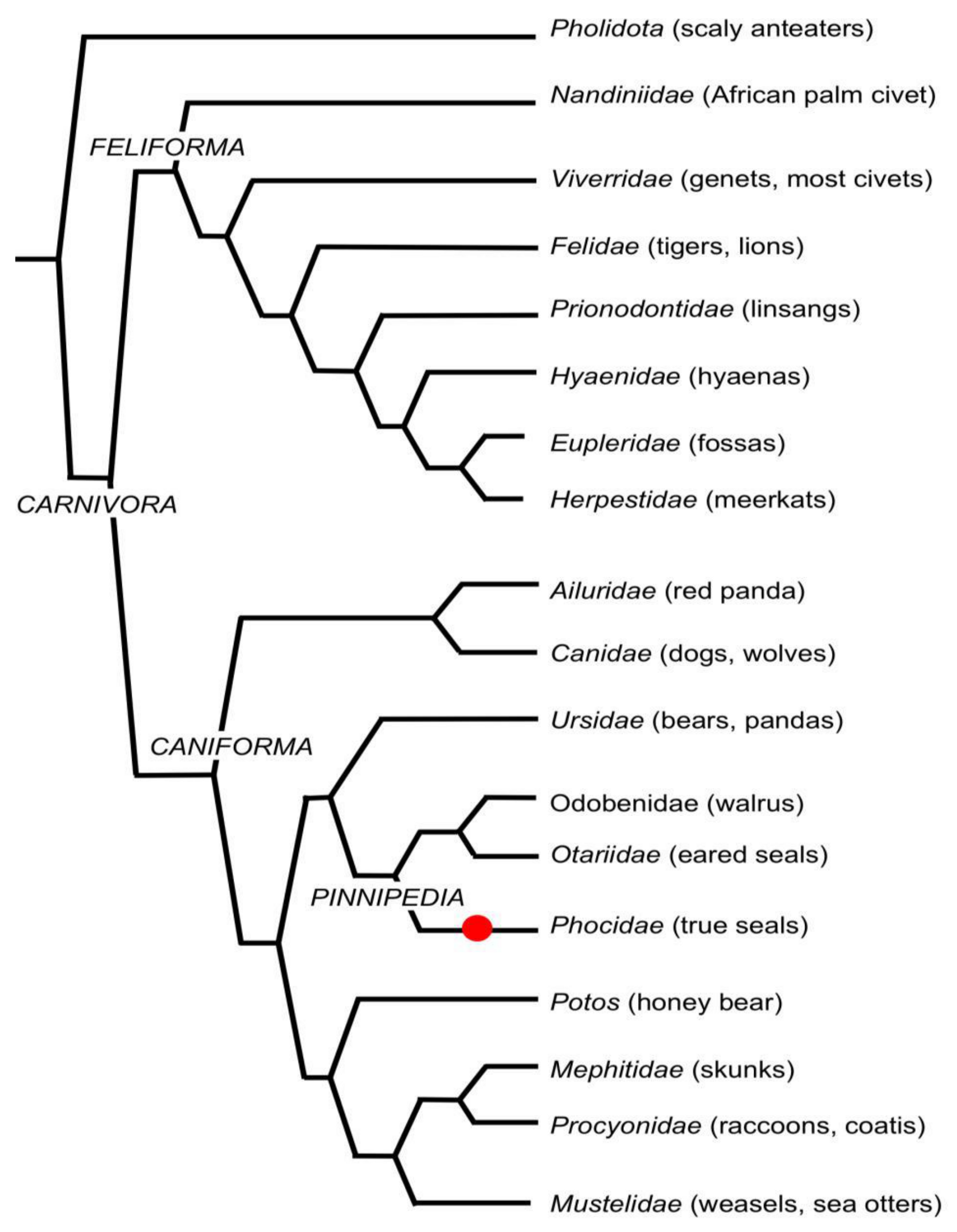

Supplement: Figure S5 — Descriptive phylogeny of families from the order Carnivora . Adapted from Agnarsson et al. 2010 [33]. Members of the Phocidae are marked with red circle. (TIF) [file pone.0083655.s005.tif]
