# Supplementary material for: The Gut Bacterial Community of Mammals from Marine and Terrestrial Habitats
Source: PLoS One. 2013 Dec 30;8(12):e83655. doi: 10.1371/journal.pone.0083655 (PMC3875473; doi:10.1371/journal.pone.0083655)
Supplement: Table S1 — Overview of main methods employed by included studies. (DOCX) [file pone.0083655.s006.docx]

**Table S1 Overview of main methods employed by included studies**

| **Study** | **DNA extraction method** | **16S rRNA gene preparation method** | **Sequencing technology/ platform** | **Primer set** |
| --- | --- | --- | --- | --- |
| [1] | Non-proprietary: See original article for details | 16S rRNA gene clone libraries | Sanger | 8/27F-1391R |
| [2] | NA | DGGE band sequencing | Sanger | NA |
| [3] | Soil DNA Extraction Kit (Mo Bio) | 16S rRNA gene clone libraries | Sanger / ABI Prism 3700 DNA Analyser (Applied Biosystems) | 8/27F-1492R |
| [4] | QIAamp Stool DNA Mini Kit (Qiagen) | 16S rRNA amplicon pyrosequencing | 454 Genome Sequencer FLX (Roche Applied Science) | 8/27F-519R |
| [5] | Non-proprietary : See original article for details | 16S rRNA amplicon pyrosequencing | 454 Genome Sequencer FLX (Roche Applied Science) | 341F-534R |
| [6] | Two methods: QIAamp Stool DNA Mini Kit (Qiagen)/ Non-proprietary: See original article for details | 16S rRNA gene clone libraries | Sanger / ABI PRISM 3100 DNA Analyser, (Applied Biosystems) | 8/27F-1492R |
| [7] | Non-proprietary: See original article for details | 16S rRNA gene clone libraries | Sanger / ABI Prism 3700 DNA Analyser (Applied Biosystems) | 8/27F-1492R |
| [8] | QIAamp Stool DNA Mini Kit (Qiagen) | 16S rRNA gene clone libraries | Sanger / “Genetic analyser” (Applied Biosystems) | 8/27F-1494R |
| [9] | Fecal DNA Isolation Kit (Mo Bio) | 16S rRNA gene clone libraries | Sanger / ABI 3730 DNA Analyser (Applied Biosystems) | U968-L1401 |
| [10] | QIAamp Stool DNA Mini Kit (Qiagen) | 16S rRNA gene clone libraries | Sanger / ABI 3730xl DNA Analyser (Applied Biosystems) | 8/27F-1391R |
| [11] | Non-proprietary : See original article for details | 16S rRNA gene clone libraries | Sanger | 8/27F-1391R |
| [12] | See original article for details | 16S rRNA gene clone libraries | Sanger / ABI Prism 3100 DNA Analyser (Applied Biosystems) | 8/27F-1492R |
| [13] | NA | 16S rRNA gene clone libraries | Sanger | NA |
| [14] | QIAamp Stool DNA Mini Kit (Qiagen) | 16S rRNA gene clone libraries | Sanger / 3130 Genetic Analyser (Applied Biosystems) | 8/27F-1494R |
| [15] | QIAamp Stool DNA Mini Kit (Qiagen) | 16S rRNA gene clone libraries | Sanger / ABI 3730 DNA Analyser (Applied Biosystems) | W02-W18 |
| [16] | QIAamp Stool DNA Mini Kit (Qiagen) | 16S rRNA gene clone libraries | Sanger / Licor sequence analyser (LI-COR Inc.) | 8/27F-1492R |
| [17] | Power Soil DNA Extraction Kit (Mo Bio) | DGGE band sequencing | Sanger / ABI 3730xl DNA Analyser (Applied Biosystems) | 968F-1401R |
| [18] | Ultra Clean Soil DNA Extraction Kit (Mo Bio) | 16S rRNA gene clone libraries | Sanger / 337 DNA Sequencer (Applied Biosystems) | 8/27F-1525R |

References

1. Ley RE, Hamady M, Lozupone C, Turnbaugh PJ, Ramey RR, et al. (2008) Evolution of mammals and their gut microbes. Science (80- ) 320: 1647–1651.

2. Wang W, Zhou Z (2009) Intestine bacteria diversity of bears in Norway. Unpublished.

3. Ozutsumi Y, Hayashi H, Sakamoto M, Itabashi H, Benno Y (2005) Culture-independent analysis of fecal microbiota in cattle. Biosci Biotechnol Biochem 69: 1793–1797.

4. Nelson TM, Rogers TL, Carlini AR, Brown M V (2012) Diet and phylogeny shape the gut microbiota of Antarctic seals: a comparison of wild and captive animals. Evol Ecol Res Cent 15: 1132–1145. Available: http://dx.doi.org/10.1111/1462-2920.12022.

5. Middelbos IS, Vester Boler BM, Qu A, White BA, Swanson KS, et al. (2010) Phylogenetic characterization of fecal microbial communities of dogs fed diets with or without supplemental dietary fiber using 454 pyrosequencing. PLoS One 5: e9768. Available: http://dx.doi.org/10.1371/journal.pone.0009768.

6. Tsukinowa E, Karita S, Asano S, Wakai Y, Oka Y, et al. (2008) Fecal microbiota of a dugong (Dugong dugong) in captivity at Toba Aquarium. J Gen Appl Microbiol 54: 25–38.

7. Nelson KE, Zinder SH, Hance I, Burr P, Odongo D, et al. (2003) Phylogenetic analysis of the microbial populations in the wild herbivore gastrointestinal tract: insights into an unexplored niche. Environ Microbiol 5: 1212–1220.

8. Glad T, Kristiansen VF, Nielsen KM, Brusetti L, Wright A-DG, et al. (2010) Ecological characterisation of the colonic microbiota in Arctic and sub-Arctic seals. Microb Ecol 60: 320–330.

9. Sonoyama K, Fujiwara R, Takemura N, Ogasawaru T, Watanabe J, et al. (2009) Response of gut microbiota to fasting and hibernation in Syrian hamsters. Appl Environ Microbiol 75: 6451–6456.

10. Eckburg PB, Bik EM, Bernstein CN, Purdom E, Dethlefsen L, et al. (2005) Diversity of the human intestinal microbial flora. Science (80- ) 308: 1635–1638. doi:10.1126/science.1110591.

11. Ley RE, Turnbaugh PJ, Klein S, Gordon JI (2006) Microbial ecology: human gut microbes associated with obesity. Nature 444: 1022–1023.

12. Hayashi H, Takahashi R, Nishi T, Sakamoto M, Benno Y (2005) Molecular analysis of jejunal, ileal, caecal and recto-sigmoidal human colonic microbiota using 16S rRNA gene libraries and terminal restriction fragment length polymorphism. J Med Microbiol 54: 1093–1101. Available: http://jmm.sgmjournals.org/cgi/content/abstract/54/11/1093.

13. Hayashi H, Sakamoto M, Benno Y (2002) Fecal microbial diversity in a strict vegetarian as determined by molecular analysis and cultivation. Microbiolgoy Immunol 46: 819–831.

14. Glad T, Bernhardsen P, Nielsen K, Brusetti L, Andersen M, et al. (2010) Bacterial diversity in faeces from polar bear (Ursus maritimus) in Arctic Svalbard. BMC Microbiol 10: 10. Available: http://www.biomedcentral.com/1471-2180/10/10.

15. Monteils V, Cauquil L, Combes S, Godon J-J, Gidenne T (2008) Potential core species and satellite species in the bacterial community within the rabbit caecum. FEMS Microbiol Ecol 66: 620–629. Available: http://dx.doi.org/10.1111/j.1574-6941.2008.00611.x.

16. Brooks SPJ, McAllister M, Sandoz M, Kalmokoff ML (2003) Culture-independent phylogenetic analysis of the faecal flora of the rat. Can J Microbiol 49: 589–601.

17. Sundset MA, Edwards JE, Cheng YF, Senosiain RS, Fraile MN, et al. (2009) Molecular diversity of the rumen microbiome of Norwegian reindeer on natural summer pasture. Microb Ecol 57: 335–348.

18. Sundset MA, Praesteng K, Cann I, Mathiesen SD, Mackie RI (2007) Novel rumen bacterial diversity in two geographically separated sub-species of reindeer. Microb Ecol 54: 424–438.
