# Supplementary material for: The Gut Bacterial Community of Mammals from Marine and Terrestrial Habitats
Source: PLoS One. 2013 Dec 30;8(12):e83655. doi: 10.1371/journal.pone.0083655 (PMC3875473; doi:10.1371/journal.pone.0083655)
Supplement: Table S2 — Characteristics of mammalian hosts used in the study. Abbreviated table data is as follows: number of sequences used (No. of seq.); gut morphology (Gut morph.); hindgut fermenter (HG); foregut fermenter (FG); simple gut (S); marine (M); terrestrial (T); carnivore (C); herbivore (H); and omnivore (O). (DOCX) [file pone.0083655.s007.docx]

**Table S2 Characteristics of mammalian hosts used in the study.**

| **Sample name** | **No. of seq.** | **Species name** | **Common name** | **Order** | **Family** | **Gut morph.** | **Habitat** | **Diet** | **Study** |
| --- | --- | --- | --- | --- | --- | --- | --- | --- | --- |
| AE1 | 92 | *Elephas maximus* | Asian Elephant | *Proboscidae* | *Elephantidae* | HG | T | H | [1] |
| AE2 | 101 | *Elephas maximus* | Asian Elephant | *Proboscidae* | *Elephantidae* | HG | T | H | [1] |
| AE3 | 97 | *Elephas maximus* | Asian Elephant | *Proboscidae* | *Elephantidae* | HG | T | H | [1] |
| AFBAB | 100 | *Papio hamadryas* | Hamadryas Baboon | *Primates* | *Cercopithecidae* | S | T | O | [1] |
| BAZ | 100 | *Papio hamadryas* | Hamadryas Baboon | *Primates* | *Cercopithecidae* | S | T | O | [1] |
| AFEL | 100 | *Loxodonta africana* | African Elephant | *Proboscidae* | *Elephantidae* | HG | T | H | [1] |
| AFEL2 | 100 | *Loxodonta africana* | African Elephant | *Proboscidae* | *Elephantidae* | HG | T | H | [1] |
| AFEL3 | 100 | *Loxodonta africana* | African Elephant | *Proboscidae* | *Elephantidae* | HG | T | H | [1] |
| AFYEL | 100 | *Loxodonta africana* | African Elephant | *Proboscidae* | *Elephantidae* | HG | T | H | [1] |
| AFZEB | 100 | *Equus zebra hartmannae* | Hartmanns Mountain Zebra | *Perissodactyla* | *Equidae* | HG | T | H | [1] |
| ARMA | 100 | *Tolypeutes matacus* | Southern Three Branded Armadillo | *Xenarthra* | *Dasypodidae* | S | T | I | [1] |
| AS1 | 100 | *Ovis ammon* | Argali Sheep | *Artiodactyla* | *Bovidae* | FG | T | H | [1] |
| AS2 | 100 | *Ovis ammon* | Argali Sheep | *Artiodactyla* | *Bovidae* | FG | T | H | [1] |
| AS3 | 100 | *Ovis ammon* | Argali Sheep | *Artiodactyla* | *Bovidae* | FG | T | H | [1] |
| BARB | 100 | *Babyrousa babyrussa* | Barbirusa | *Artiodactyla* | *Suidae* | FG | T | O | [1] |
| BAT | 100 | *Carollia perspicillata* | Sebas Short-tailed Bat | *Chiroptera* | *Phyllostomidae* | S | T | O | [1] |
| BB1 | 100 | *Ursus americanus* | North American Black Bear | *Carnivora* | *Ursidae* | S | T | O | [1] |
| BB2 | 100 | *Ursus americanus* | North American Black Bear | *Carnivora* | *Ursidae* | S | T | O | [1] |
| BDOG1 | 100 | *Speothos venaticus* | Bushdog | *Carnivora* | *Canidae* | S | T | C | [1] |
| BDOG3 | 34 | *Speothos venaticus* | Bushdog | *Carnivora* | *Canidae* | S | T | C | [1] |
| BEAR | 40 | *Ursus sp.* | Bear from Norway | *Carnivora* | *Ursidae* | S | T | O | [2] |
| BG | 97 | *Bos javanicus* | Banteng | *Artiodactyla* | *Bovidae* | FG | T | H | [1] |
| BH1 | 100 | *Ovis canadensis* | Bighorn Sheep | *Artiodactyla* | *Bovidae* | FG | T | H | [1] |
| BH2 | 100 | *Ovis canadensis* | Bighorn Sheep | *Artiodactyla* | *Bovidae* | FG | T | H | [1] |
| BHSD | 100 | *Ovis canadensis* | Bighorn Sheep | *Artiodactyla* | *Bovidae* | FG | T | H | [1] |
| BKLE | 100 | *Eulemur macaco* | Black Lemur | *Primates* | *Lemuridae* | S | T | O | [1] |
| BNO | 88 | *Pan paniscus* | Bonobo | *Primates* | *Hominidae* | S | T | O | [1] |
| CAL | 100 | *Callimico goeldii* | Goeldis Marmoset | *Primates* | *Cebidae* | S | T | O | [1] |
| CAP | 100 | *Hydrochoerus hydrochaeris* | Capybara | *Rodentia* | *Caviidae* | HG | T | H | [1] |
| CATT | 100 | *Bos taurus* | Holstein Cattle | *Artiodactyla* | *Bovidae* | FG | T | H | [3] |
| CE2 | 100 | *Acinonyx jubatus* | Cheetah | *Carnivora* | *Felidae* | S | T | C | [1] |
| CE3 | 90 | *Acinonyx jubatus* | Cheetah | *Carnivora* | *Felidae* | S | T | C | [1] |
| CHIMP1 | 100 | *Pan troglodytes* | Chimpanzee | *Primates* | *Hominidae* | S | T | O | [1] |
| CHIMP12 | 80 | *Pan troglodytes* | Chimpanzee | *Primates* | *Hominidae* | S | T | O | [1] |
| CLS1 | 100 | *Hydrurga leptonyx* | Leopard Seal | *Carnivora* | *Phocidae* | S | M | CM | [4] |
| CLS2 | 100 | *Hydrurga leptonyx* | Leopard Seal | *Carnivora* | *Phocidae* | S | M | CM | [4] |
| COL | 101 | *Colobus guereza kikuyuensis* | Eastern Black and White Colobus | *Primates* | *Cercopithecidae* | FG | T | H | [1] |
| DL | 100 | *Pygathrix sp.* | Douc Langur | *Primates* | *Cercopithecidae* | FG | T | H | [1] |
| DOG_ | 100 | *Canis lupus familiaris* | Dog | *Carnivora* | *Canidae* | S | T | O | [5] |
| DOG | 100 | *Canis lupus familiaris* | Dog | *Carnivora* | *Canidae* | S | T | O | [5] |
| DOG | 100 | *Canis lupus familiaris* | Dog | *Carnivora* | *Canidae* | S | T | O | [5] |
| DOG | 100 | *Canis lupus familiaris* | Dog | *Carnivora* | *Canidae* | S | T | O | [5] |
| DOG | 100 | *Canis lupus familiaris* | Dog | *Carnivora* | *Canidae* | S | T | O | [5] |
| DUG | 90 | *Dugong dugong* | Dugong | *Sirenia* | *Dugonidae* |  | M | HM | [6] |
| EAC | 100 | *Colobus angolensis* | East Angolan Colobus | *Primates* | *Cercopithecidae* | FG | T | H | [1] |
| ECH | 100 | *Tachyglossus aculeatus* | Short Beaked Echidna | *Monotremata* | *Tachyglossidae* | S | T | CI | [1] |
| ELAND | 64 | *Taurotragus oryx* | Eland | *Artiodactyla* | *Bovidae* | FG | T | H | [7] |
| ES01 | 100 | *Mirounga leonina* | Southern Elephant Seal | *Carnivora* | *Phocidae* | S | M | CM | [4] |
| ES04 | 100 | *Mirounga leonina* | Southern Elephant Seal | *Carnivora* | *Phocidae* | S | M | CM | [4] |
| ES10 | 100 | *Mirounga leonina* | Southern Elephant Seal | *Carnivora* | *Phocidae* | S | M | CM | [4] |
| ES11 | 100 | *Mirounga leonina* | Southern Elephant Seal | *Carnivora* | *Phocidae* | S | M | CM | [4] |
| ES12 | 100 | *Mirounga leonina* | Southern Elephant Seal | *Carnivora* | *Phocidae* | S | M | CM | [4] |
| ES14 | 100 | *Mirounga leonina* | Southern Elephant Seal | *Carnivora* | *Phocidae* | S | M | CM | [4] |
| ES15 | 100 | *Mirounga leonina* | Southern Elephant Seal | *Carnivora* | *Phocidae* | S | M | CM | [4] |
| ES16 | 100 | *Mirounga leonina* | Southern Elephant Seal | *Carnivora* | *Phocidae* | S | M | CM | [4] |
| ES17 | 100 | *Mirounga leonina* | Southern Elephant Seal | *Carnivora* | *Phocidae* | S | M | CM | [4] |
| ES20 | 100 | *Mirounga leonina* | Southern Elephant Seal | *Carnivora* | *Phocidae* | S | M | CM | [4] |
| ES21 | 100 | *Mirounga leonina* | Southern Elephant Seal | *Carnivora* | *Phocidae* | S | M | CM | [4] |
| ES22 | 100 | *Mirounga leonina* | Southern Elephant Seal | *Carnivora* | *Phocidae* | S | M | CM | [4] |
| ES24 | 100 | *Mirounga leonina* | Southern Elephant Seal | *Carnivora* | *Phocidae* | S | M | CM | [4] |
| ES25 | 100 | *Mirounga leonina* | Southern Elephant Seal | *Carnivora* | *Phocidae* | S | M | CM | [4] |
| ES26 | 100 | *Mirounga leonina* | Southern Elephant Seal | *Carnivora* | *Phocidae* | S | M | CM | [4] |
| ES28 | 100 | *Mirounga leonina* | Southern Elephant Seal | *Carnivora* | *Phocidae* | S | M | CM | [4] |
| ES29 | 100 | *Mirounga leonina* | Southern Elephant Seal | *Carnivora* | *Phocidae* | S | M | CM | [4] |
| ES30 | 100 | *Mirounga leonina* | Southern Elephant Seal | *Carnivora* | *Phocidae* | S | M | CM | [4] |
| ES31 | 100 | *Mirounga leonina* | Southern Elephant Seal | *Carnivora* | *Phocidae* | S | M | CM | [4] |
| ES33 | 100 | *Mirounga leonina* | Southern Elephant Seal | *Carnivora* | *Phocidae* | S | M | CM | [4] |
| ES34 | 100 | *Mirounga leonina* | Southern Elephant Seal | *Carnivora* | *Phocidae* | S | M | CM | [4] |
| ES37 | 100 | *Mirounga leonina* | Southern Elephant Seal | *Carnivora* | *Phocidae* | S | M | CM | [4] |
| ES40 | 100 | *Mirounga leonina* | Southern Elephant Seal | *Carnivora* | *Phocidae* | S | M | CM | [4] |
| ES45 | 100 | *Mirounga leonina* | Southern Elephant Seal | *Carnivora* | *Phocidae* | S | M | CM | [4] |
| FF | 100 | *Pteropus scapulatus* | Flying Fox | *Chiroptera* | *Pterodidae* | S | T | H | [1] |
| FL | 100 | *Trachypithecus francoisi* | Francois Langur | *Primates* | *Cercopithecidae* | FG | T | H | [1] |
| GAZ_GRA | 60 | *Nanger granti* | Grants Gazelle | *Artiodactyla* | *Bovidae* | FG | T | H | [7] |
| GAZ_THO | 77 | *Eudorcas thomsonii* | Thomsons Gazelle | *Artiodactyla* | *Bovidae* | FG | T | H | [7] |
| GIR | 100 | *Giraffa camelopardalis reticulata* | Reticulated Giraffe | *Artiodactyla* | *Giraffidae* | FG | T | H | [1] |
| GOR | 100 | *Gorilla gorilla gorilla* | Western Lowland Gorilla | *Primates* | *Hominidae* | HG | T | H | [1] |
| GORSD | 100 | *Gorilla gorilla gorilla* | Western Lowland Gorilla | *Primates* | *Hominidae* | HG | T | H | [1] |
| GP | 100 | *Ailuropoda melanoleuca* | Giant Panda | *Carnivora* | *Ursidae* | S | T | H | [1] |
| GREY_GL | 76 | *Halichoerus grypus* | Grey Seal | *Carnivora* | *Phocidae* | S | M | CM | [8] |
| GZ | 100 | *Equus grevyi* | Grevys Zebra | *Perissodactyla* | *Equidae* | HG | T | H | [1] |
| HAMS | 100 | *Mesocricetus auratus* | Hamster | *Rodentia* | *Cricetidae* | HG | T | O | [9] |
| HARB_GL | 77 | *Phoca vitulina* | Harbour Seal | *Carnivora* | *Phocidae* | S | M | CM | [8] |
| HH | 100 | *Erinaceus albiventris* | Hedgehog | *Insectivora* | *Erinaceidae* | S | T | CI | [1] |
| HOOD_GL | 100 | *Cystophora cristata* | Hooded Seal | *Carnivora* | *Phocidae* | S | M | CM | [8] |
| HORSEJ | 100 | *Equus ferus caballus* | Horse | *Perissodactyla* | *Equidae* | HG | T | H | [1] |
| HORSEM | 100 | *Equus ferus caballus* | Horse | *Perissodactyla* | *Equidae* | HG | T | H | [1] |
| HRX | 100 | *Procavia capensis* | Rock Hyrax | *Hyracoidea* | *Procaviidae* | FG | T | H | [1] |
| HUM_ECK | 100 | *Homo sapiens sapiens* | Human | *Primates* | *Hominidae* | S | T | O | [10] |
| HUM_FAT | 100 | *Homo sapiens sapiens* | Human | *Primates* | *Hominidae* | S | T | O | [11] |
| HUM_OLD | 88 | *Homo sapiens sapiens* | Human | *Primates* | *Hominidae* | S | T | O | [12] |
| HUM_VEG | 61 | *Homo sapiens sapiens* | Human | *Primates* | *Hominidae* | S | T | O | [13] |
| HY1 | 100 | *Crocuta crocuta* | Spotted Hyena | *Carnivora* | *Hyaenidae* | S | T | C | [1] |
| HY2 | 100 | *Crocuta crocuta* | Spotted Hyena | *Carnivora* | *Hyaenidae* | S | T | C | [1] |
| IR | 100 | *Rhinoceros unicornis* | Indian Rhino | *Perissodactyla* | *Rhinocerotidae* | HG | T | H | [1] |
| KO1 | 100 | *Macropus rufus* | Red Kangaroo | *Diprotodontia* | *Macropidae* | FG | T | H | [1] |
| KO2 | 97 | *Macropus rufus* | Red Kangaroo | *Diprotodontia* | *Macropidae* | FG | T | H | [1] |
| LI1 | 80 | *Panthera leo* | Lion | *Carnivora* | *Pantherinae* | S | T | C | [1] |
| LI2 | 100 | *Panthera leo* | Lion | *Carnivora* | *Pantherinae* | S | T | C | [1] |
| LI3 | 100 | *Panthera leo* | Lion | *Carnivora* | *Pantherinae* | S | T | C | [1] |
| MAR | 100 | *Callithrix geoffroyi* | Geoffreys Marmoset | *Primates* | *Callitrichidae* | S | T | O | [1] |
| ML | 100 | *Eulemur mongoz* | Mongoose Lemur | *Primates* | *Lemuridae* | S | T | O | [1] |
| MOLERAT | 102 | *Heterocephalus glaber* | Naked Molerat | *Rodentia* | *Bathyergidae* | HG | T | H | [1] |
| OK1 | 100 | *Okapia johnstoni* | Okapi | *Artiodactyla* | *Giraffidae* | FG | T | H | [1] |
| OK2 | 100 | *Okapia johnstoni* | Okapi | *Artiodactyla* | *Giraffidae* | FG | T | H | [1] |
| OK3 | 100 | *Okapia johnstoni* | Okapi | *Artiodactyla* | *Giraffidae* | FG | T | H | [1] |
| ORANG1 | 100 | *Pongo pygmaeus* | Orangutan | *Primates* | *Hominidae* | HG | T | H | [1] |
| ORANG2 | 100 | *Pongo pygmaeus* | Orangutan | *Primates* | *Hominidae* | HG | T | H | [1] |
| PB1 | 100 | *Ursus maritimus* | Polar Bear | *Carnivora* | *Ursidae* | S | M | CM | [1] |
| PB2 | 100 | *Ursus maritimus* | Polar Bear | *Carnivora* | *Ursidae* | S | M | CM | [1] |
| PBF_GL | 100 | *Ursus maritimus* | Polar Bear | *Carnivora* | *Ursidae* | S | M | CM | [14] |
| PBM_GL | 24 | *Ursus maritimus* | Polar Bear | *Carnivora* | *Ursidae* | S | M | CM | [14] |
| RA | 100 | *Oryctolagus cuniculus* | European Rabbit | *Lagomorpha* | *Leporidae* | HG | T | H | [1] |
| RABB | 100 | *Oryctolagus cuniculus* | European Rabbit | *Lagomorpha* | *Leporidae* | HG | T | H | [15] |
| RAT | 80 | *Rattus norvegicus* | Norway Rat (Wistar) | *Rodentia* | *Muridae* | HG | T | O | [16] |
| REIN_NORNAT | 34 | *Rangifer tarandus* | Norway Reindeer | *Artiodactyla* | *Cervidae* | FG | T | H | [17] |
| REIN_NORPEL | 57 | *Rangifer tarandus* | Norway Reindeer | *Artiodactyla* | *Cervidae* | FG | T | H | [17] |
| REIN_SVAL | 31 | *Rangifer tarandus platyrhynchus* | Svalbard Reindeer | *Artiodactyla* | *Cervidae* | FG | T | H | [18] |
| RH | 100 | *Diceros bicornis* | Black Rhino | *Perissodactyla* | *Rhinocerotidae* | HG | T | H | [1] |
| RHSD | 100 | *Procavia capensis* | Rock Hyrax | *Hyracoidea* | *Procaviidae* | FG | T | H | [1] |
| RP | 100 | *Ailurus fulgens* | Red Panda | *Carnivora* | *Ailuridae* | S | T | H | [1] |
| RPSD | 100 | *Ailurus fulgens* | Red Panda | *Carnivora* | *Ailuridae* | S | T | H | [1] |
| RRH | 100 | *Potamochoerus porcus* | Red River Hog | *Artiodactyla* | *Suidae* | FG | T | O | [1] |
| RT | 100 | *Lemur catta* | Ring Tailed Lemur | *Primates* | *Lemuridae* | S | T | O | [1] |
| SAKI | 100 | *Pithecia pithecia* | White Faced Saki | *Primates* | *Pitheciidae* | S | T | O | [1] |
| SB | 100 | *Tremarctos ornatus* | Spectacled Bear | *Carnivora* | *Ursidae* | S | T | O | [1] |
| SBK | 32 | *Antidorcas marsupialis* | Springbok | *Artiodactyla* | *Bovidae* | FG | T | H | [1] |
| SBSD | 100 | *Antidorcas marsupialis* | Springbok | *Artiodactyla* | *Bovidae* | FG | T | H | [1] |
| SP2 | 100 | *Gazella spekei* | Spekes Gazelle | *Artiodactyla* | *Bovidae* | FG | T | H | [1] |
| SP3 | 100 | *Gazella spekei* | Spekes Gazelle | *Artiodactyla* | *Bovidae* | FG | T | H | [1] |
| SPIM | 100 | *Ateles geoffroyi* | Black Handed Spider Monkey | *Primates* | *Atelidae* | S | T | O | [1] |
| SQ | 100 | *Callosciurus prevostii* | Prevosts Squirrel | *Rodentia* | *Sciuridae* | S | T | O | [1] |
| TAK | 100 | *Budorcas taxicolor* | Takin | *Artiodactyla* | *Bovidae* | FG | T | H | [1] |
| TU1 | 100 | *Ovis orientalis arkal* | Transcaspian Urial Sheep | *Artiodactyla* | *Bovidae* | FG | T | H | [1] |
| TU2 | 100 | *Ovis orientalis arkal* | Transcaspian Urial Sheep | *Artiodactyla* | *Bovidae* | FG | T | H | [1] |
| VWP | 100 | *Sus cebifrons* | Visayan Warty Pig | *Artiodactyla* | *Suidae* | FG | T | H | [1] |
| WA | 101 | *Equus africanus somaliensis* | Somali Wild Ass | *Perissodactyla* | *Equidae* | HG | T | H | [1] |
| WLS49 | 100 | *Hydrurga leptonyx* | Leopard Seal | *Carnivora* | *Phocidae* | S | M | CM | [4] |
| WLS50 | 100 | *Hydrurga leptonyx* | Leopard Seal | *Carnivora* | *Phocidae* | S | M | CM | [4] |
| WLS51 | 100 | *Hydrurga leptonyx* | Leopard Seal | *Carnivora* | *Phocidae* | S | M | CM | [4] |
| WLS52 | 100 | *Hydrurga leptonyx* | Leopard Seal | *Carnivora* | *Phocidae* | S | M | CM | [4] |
| WLS53 | 100 | *Hydrurga leptonyx* | Leopard Seal | *Carnivora* | *Phocidae* | S | M | CM | [4] |
| WLS54 | 100 | *Hydrurga leptonyx* | Leopard Seal | *Carnivora* | *Phocidae* | S | M | CM | [4] |
| WLS56 | 100 | *Hydrurga leptonyx* | Leopard Seal | *Carnivora* | *Phocidae* | S | M | CM | [4] |
| WLS57 | 100 | *Hydrurga leptonyx* | Leopard Seal | *Carnivora* | *Phocidae* | S | M | CM | [4] |
| WLS58 | 100 | *Hydrurga leptonyx* | Leopard Seal | *Carnivora* | *Phocidae* | S | M | CM | [4] |
| WLS59 | 100 | *Hydrurga leptonyx* | Leopard Seal | *Carnivora* | *Phocidae* | S | M | CM | [4] |
| WLS60 | 100 | *Hydrurga leptonyx* | Leopard Seal | *Carnivora* | *Phocidae* | S | M | CM | [4] |
| WLS63 | 100 | *Hydrurga leptonyx* | Leopard Seal | *Carnivora* | *Phocidae* | S | M | CM | [4] |
| ZEB_NELS | 41 | *Equus quagga* | Plains Zebra | *Perissodactyla* | *Equidae* | HG | T | H | [7] |
| ZEBU | 30 | *Bos primigenius* | African Zebu Cattle | *Artiodactyla* | *Bovidae* | FG | T | H | [7] |

Abbreviated table data is as follows: number of sequences used (No. of seq.); gut morphology (Gut morph.); hindgut fermenter (HG); foregut fermenter (FG); simple gut (S); marine (M); terrestrial (T); carnivore (C); herbivore (H); and omnivore (O).

**References**

1. Ley RE, Hamady M, Lozupone C, Turnbaugh PJ, Ramey RR, et al. (2008) Evolution of mammals and their gut microbes. Science (80- ) 320: 1647–1651.

2. Wang W, Zhou Z (2009) Intestine bacteria diversity of bears in Norway. Unpublished.

3. Ozutsumi Y, Hayashi H, Sakamoto M, Itabashi H, Benno Y (2005) Culture-independent analysis of fecal microbiota in cattle. Biosci Biotechnol Biochem 69: 1793–1797.

4. Nelson TM, Rogers TL, Carlini AR, Brown M V (2012) Diet and phylogeny shape the gut microbiota of Antarctic seals: a comparison of wild and captive animals. Evol Ecol Res Cent 15: 1132–1145. Available: http://dx.doi.org/10.1111/1462-2920.12022.

5. Middelbos IS, Vester Boler BM, Qu A, White BA, Swanson KS, et al. (2010) Phylogenetic characterization of fecal microbial communities of dogs fed diets with or without supplemental dietary fiber using 454 pyrosequencing. PLoS One 5: e9768. Available: http://dx.doi.org/10.1371/journal.pone.0009768.

6. Tsukinowa E, Karita S, Asano S, Wakai Y, Oka Y, et al. (2008) Fecal microbiota of a dugong (Dugong dugong) in captivity at Toba Aquarium. J Gen Appl Microbiol 54: 25–38.

7. Nelson KE, Zinder SH, Hance I, Burr P, Odongo D, et al. (2003) Phylogenetic analysis of the microbial populations in the wild herbivore gastrointestinal tract: insights into an unexplored niche. Environ Microbiol 5: 1212–1220.

8. Glad T, Kristiansen VF, Nielsen KM, Brusetti L, Wright A-DG, et al. (2010) Ecological characterisation of the colonic microbiota in Arctic and sub-Arctic seals. Microb Ecol 60: 320–330.

9. Sonoyama K, Fujiwara R, Takemura N, Ogasawaru T, Watanabe J, et al. (2009) Response of gut microbiota to fasting and hibernation in Syrian hamsters. Appl Environ Microbiol 75: 6451–6456.

10. Eckburg PB, Bik EM, Bernstein CN, Purdom E, Dethlefsen L, et al. (2005) Diversity of the human intestinal microbial flora. Science (80- ) 308: 1635–1638. doi:10.1126/science.1110591.

11. Ley RE, Turnbaugh PJ, Klein S, Gordon JI (2006) Microbial ecology: human gut microbes associated with obesity. Nature 444: 1022–1023.

12. Hayashi H, Takahashi R, Nishi T, Sakamoto M, Benno Y (2005) Molecular analysis of jejunal, ileal, caecal and recto-sigmoidal human colonic microbiota using 16S rRNA gene libraries and terminal restriction fragment length polymorphism. J Med Microbiol 54: 1093–1101. Available: http://jmm.sgmjournals.org/cgi/content/abstract/54/11/1093.

13. Hayashi H, Sakamoto M, Benno Y (2002) Fecal microbial diversity in a strict vegetarian as determined by molecular analysis and cultivation. Microbiolgoy Immunol 46: 819–831.

14. Glad T, Bernhardsen P, Nielsen K, Brusetti L, Andersen M, et al. (2010) Bacterial diversity in faeces from polar bear (Ursus maritimus) in Arctic Svalbard. BMC Microbiol 10: 10. Available: http://www.biomedcentral.com/1471-2180/10/10.

15. Monteils V, Cauquil L, Combes S, Godon J-J, Gidenne T (2008) Potential core species and satellite species in the bacterial community within the rabbit caecum. FEMS Microbiol Ecol 66: 620–629. Available: http://dx.doi.org/10.1111/j.1574-6941.2008.00611.x.

16. Brooks SPJ, McAllister M, Sandoz M, Kalmokoff ML (2003) Culture-independent phylogenetic analysis of the faecal flora of the rat. Can J Microbiol 49: 589–601.

17. Sundset MA, Edwards JE, Cheng YF, Senosiain RS, Fraile MN, et al. (2009) Molecular diversity of the rumen microbiome of Norwegian reindeer on natural summer pasture. Microb Ecol 57: 335–348.

18. Sundset MA, Praesteng K, Cann I, Mathiesen SD, Mackie RI (2007) Novel rumen bacterial diversity in two geographically separated sub-species of reindeer. Microb Ecol 54: 424–438.
