# Supplementary material for: The Gut Bacterial Community of Mammals from Marine and Terrestrial Habitats
Source: PLoS One. 2013 Dec 30;8(12):e83655. doi: 10.1371/journal.pone.0083655 (PMC3875473; doi:10.1371/journal.pone.0083655)
Supplement: Table S3 — Comparison of ANOSIM results between the genera rarefied to the minimum of 24 and those subsampled to 100. Results display those reported in Table 1 and the comparable results when the dataset was rarefied to the minimum number of sequences represented by any one host which was 24. ANOSIM of gut bacterial abundance data was used to generate a permutated Global R statistic (R) and permutated p-value (p). Significance level: **R = >0.5, *R = 0.3< R <0.5. (DOCX) [file pone.0083655.s008.docx]

Table S3 Comparison of ANOSIM results between the genera rarefied to the minimum of 24 and those subsampled to 100.

| **Source of variation** | **Pair-wise comparisons** | ***R (Table1)*** | ***P (Table1)*** | ***R*** | ***p*** |
| --- | --- | --- | --- | --- | --- |
| Diet |  | 0.39* | < 0.01 | 0.34* | < 0.01 |
|  | Herbivores, omnivores | 0.33* | < 0.01 | 0.29 | < 0.01 |
|  | Herbivores, carnivores | 0.49* | < 0.01 | 0.43* | < 0.01 |
|  | Omnivores, carnivores | 0.26 | < 0.01 | 0.24 | < 0.01 |
| Diet and habitat | | 0.52** | < 0.01 | 0.44* | < 0.01 |
|  | Terrestrial herbivores, terrestrial omnivores | 0.33* | < 0.01 | 0.29 | < 0.01 |
|  | Terrestrial herbivores, terrestrial carnivores | 0.62** | < 0.01 | 0.45* | < 0.01 |
|  | Terrestrial herbivores, marine carnivores | 0.65** | < 0.01 | 0.56** | < 0.01 |
|  | Terrestrial omnivores, terrestrial carnivore | 0.32 | < 0.01 | 0.22 | < 0.01 |
|  | Terrestrial omnivores, marine carnivores | 0.50** | < 0.01 | 0.43* | < 0.01 |
|  | Terrestrial carnivores, marine carnivores | 0.69** | < 0.01 | 0.59** | < 0.01 |
| Phylogenetic order | | 0.19 | < 0.01 | 0.24 | < 0.01 |
| Phylogenetic family | | 0.50** | < 0.01 | 0.47* | < 0.01 |
| Gut morphology | | 0.11 | < 0.01 | 0.12 | < 0.01 |
|  | Hindgut fermentors, simple guts | 0.14 | < 0.01 | 0.16 | < 0.01 |
|  | Hindgut fermentors, foregut fermentors | 0.17 | < 0.01 | 0.15 | < 0.01 |
|  | Simple guts, foregut fermentors | 0.15 | < 0.01 | 0.20 | < 0.01 |

Results display those reported in Table 1 and the comparable results when the dataset was rarefied to the minimum number of sequences represented by any one host which was 24. ANOSIM of gut bacterial abundance data was used to generate a permutated Global R statistic (R) and permutated p-value (*p*). Significance level: **R = > 0.5, *R = 0.3 < R < 0.5.
