# Supplementary material for: The Gut Bacterial Community of Mammals from Marine and Terrestrial Habitats
Source: PLoS One. 2013 Dec 30;8(12):e83655. doi: 10.1371/journal.pone.0083655 (PMC3875473; doi:10.1371/journal.pone.0083655)
Supplement: Table S4 — Characteristic genera in the gut bacterial community of mammal hosts grouped by diet and habitat. The foremost ten characteristic genera in the gut bacterial community of hosts identified using SIMPER analysis. Hosts are grouped based on diet and habitat. (DOCX) [file pone.0083655.s009.docx]

**Table S4 Characteristic genera in the gut bacterial community of mammal hosts grouped by diet and habitat.**

| **Group** | **Phylum** | **Genus** | **Average abundance** | **Contribution to total (%)** |
| --- | --- | --- | --- | --- |
| **Terrestrial herbivores** | *Firmicutes* | *Oscillibacter* | 2.4 | 10.4 |
|  | *Firmicutes* | *Coprococcus* | 2.0 | 9.4 |
|  | *Bacteroidetes* | *Rikenella* | 1.6 | 6.8 |
|  | *Firmicutes* | *Papillibacter* | 1.5 | 6.4 |
|  | *Firmicutes* | *Robinsoniella* | 1.3 | 4.9 |
|  | *Firmicutes* | *Acetivibrio* | 1.3 | 4.7 |
|  | *Firmicutes* | *Sporobacter* | 1.2 | 4.6 |
|  | *Firmicutes* | *Ruminococcus* | 1.3 | 4.5 |
|  | *Firmicutes* | *Ethanoligenens* | 1.0 | 3.2 |
|  | *Firmicutes* | *Anaerotruncus* | 0.9 | 3.0 |
| **Terrestrial omnivores** | *Bacteroidetes* | *Prevotella* | 2.2 | 11.9 |
|  | *Firmicutes* | *Coprococcus* | 1.7 | 10.9 |
|  | *Firmicutes* | *Blautia* | 1.9 | 10.4 |
|  | *Firmicutes* | *Streptococcus* | 1.5 | 4.8 |
|  | *Firmicutes* | *Oscillibacter* | 1.2 | 4.6 |
|  | *Bacteroidetes* | *Bacteroides* | 1.5 | 4.5 |
|  | *Firmicutes* | *Robinsoniella* | 0.9 | 3.8 |
|  | *Firmicutes* | *Faecalibacterium* | 1.0 | 3.6 |
|  | *Bacteroidetes* | *Barnesiella* | 0.9 | 3.5 |
|  | *Proteobacteria* | *Hallella* | 0.7 | 3.3 |
| **Terrestrial carnivores** | *Firmicutes* | *Peptostreptococcus* | 3.1 | 19.3 |
|  | *Firmicutes* | *Clostridium* | 2.7 | 16.6 |
|  | *Firmicutes* | *Sporacetigenium* | 2.1 | 11.7 |
|  | *Firmicutes* | *Blautia* | 2.4 | 11.5 |
|  | *Firmicutes* | *Coprococcus* | 1.5 | 7.8 |
|  | *Actinobacteria* | *Collinsella* | 1.5 | 5.7 |
|  | *Firmicutes* | *Lactobacillus* | 1.8 | 5.6 |
|  | *Proteobacteria* | *Escherichia / Shigella* | 1.2 | 5.0 |
|  | *Firmicutes* | *Robinsoniella* | 0.8 | 2.6 |
|  | *Firmicutes* | *Enterococcus* | 0.8 | 2.5 |
| **Marine carnivores** | *Fusobacteria* | *Fusobacterium* | 3.0 | 16.8 |
|  | *Firmicutes* | *Faecalibacterium* | 1.9 | 9.8 |
|  | *Fusobacteria* | *Cetobacterium* | 1.9 | 8.9 |
|  | *Firmicutes* | *Oscillibacter* | 1.6 | 7.4 |
|  | *Proteobacteria* | *Psychrobacter* | 1.9 | 6.2 |
|  | *Bacteroidetes* | *Bacteroides* | 1.5 | 5.5 |
|  | *Firmicutes* | *Butyricicoccus* | 0.9 | 3.6 |
|  | *Firmicutes* | *Sporanaerobacter* | 1.0 | 3.2 |
|  | *Firmicutes* | *Sporobacter* | 0.7 | 3.0 |
|  | *Bacteroidetes* | *Porphyromonas* | 1.1 | 2.9 |
| **Marine herbivore** | *Firmicutes* | *Clostridium* | 10.0 | 11.1 |
|  | *Firmicutes* | *Coprococcus* | 7.0 | 7.8 |
|  | *Bacteroidetes* | *Bacteroides* | 6.0 | 6.7 |
|  | *Bacteroidetes* | *Prevotella* | 6.0 | 6.7 |
|  | *Firmicutes* | *Oscillibacter* | 5.0 | 5.6 |
|  | *Verrucomicrobia* | *Akkermansia* | 4.0 | 4.4 |
|  | *Firmicutes* | *Anaerotruncus* | 4.0 | 4.4 |
|  | *Bacteroidetes* | *Alistipes* | 3.0 | 3.3 |
|  | *Firmicutes* | *Roseburia* | 3.0 | 3.3 |
|  | *Firmicutes* | *Ruminococcus* | 3.0 | 3.3 |

The foremost ten characteristic genera in the gut bacterial community of hosts identified using SIMPER analysis. Hosts are grouped based on diet and habitat.
